# The Effects of Stimulus Variability on the Perceptual Learning of Speech and Non-Speech Stimuli

**DOI:** 10.1371/journal.pone.0118465

**Published:** 2015-02-25

**Authors:** Karen Banai, Sygal Amitay

**Affiliations:** 1 Department of Communication Sciences and Disorders, University of Haifa, Haifa, Israel; 2 Medical Research Council—Institute of Hearing Research, Nottingham, United Kingdom; Harvard Medical School/Massachusetts General Hospital, UNITED STATES

## Abstract

Previous studies suggest fundamental differences between the perceptual learning of speech and non-speech stimuli. One major difference is in the way variability in the training set affects learning and its generalization to untrained stimuli: training-set variability appears to facilitate speech learning, while slowing or altogether extinguishing non-speech auditory learning. We asked whether the reason for this apparent difference is a consequence of the very different methodologies used in speech and non-speech studies. We hypothesized that speech and non-speech training would result in a similar pattern of learning if they were trained using the same training regimen. We used a 2 (random vs. blocked pre- and post-testing) × 2 (random vs. blocked training) × 2 (speech vs. non-speech discrimination task) study design, yielding 8 training groups. A further 2 groups acted as untrained controls, tested with either random or blocked stimuli. The speech task required syllable discrimination along 4 minimal-pair continua (e.g., bee-dee), and the non-speech stimuli required duration discrimination around 4 base durations (e.g., 50 ms). Training and testing required listeners to pick the odd-one-out of three stimuli, two of which were the base duration or phoneme continuum endpoint and the third varied adaptively. Training was administered in 9 sessions of 640 trials each, spread over 4–8 weeks. Significant learning was only observed following speech training, with similar learning rates and full generalization regardless of whether training used random or blocked schedules. No learning was observed for duration discrimination with either training regimen. We therefore conclude that the two stimulus classes respond differently to the same training regimen. A reasonable interpretation of the findings is that speech is perceived categorically, enabling learning in either paradigm, while the different base durations are not well-enough differentiated to allow for categorization, resulting in disruption to learning.

## Introduction

Despite decades of research in perceptual learning, it is not clear whether the perceptual learning of the acoustic elements of speech [1] and non-speech [2, 3] sounds are based on the same underlying mechanisms. These two forms of perceptual learning were generally studied separately, by different communities of investigators with greatly varying goals and methodologies. One striking difference between these two bodies of research, which is the focus of the present study, is in the putative role of stimulus variability in learning (see [4] for review). On the one hand, the perceptual learning of non-speech acoustic elements is most often studied with training regimens that involve massive repetition of the same stimulus tokens throughout training (e.g., [5, 6]). In the rare cases in which stimulus variability was introduced by the presentation of several randomly mixed stimuli during training (roving), the effect seemed detrimental to learning [7, 8]. On the other hand, the perceptual learning of speech was predominantly studied with regimens that involve little or no stimulus repetition throughout training [9, 10]. In these studies, when stimulus variability was limited by the introduction of greater stimulus repetition, generalization to untrained tokens seemed to diminish [9–12]. The goal of the current study was to directly compare the effects of stimulus variability on the learning of speech and non-speech elements. For reasons discussed below, we theorize that the differential effects of stimulus variability are related to the relevance of the variable dimension to learning, rather than to true differences in learning mechanisms between the two domains. Three specific hypotheses derived from this idea were tested in this study:

H1: Randomly varying the stimuli along a training-relevant dimension and presenting them in blocks (keeping them constant on a trial-by-trial basis) will result in similar amounts of learning.
H2: Stimulus variability slows learning.
H3: The effects of variability are similar in the learning of speech and non-speech elements once training and testing conditions are equated across the two types of stimuli.

A comparison of the outcomes of perceptual training with speech and non-speech stimuli suggests that in general, greater effects of training are observed after training with speech stimuli than after training with non-speech ones because wider generalization to untrained tokens occurs with speech than with non-speech training [4]. For example, multiday training on speech identification under adverse listening conditions (noise, time-compression, a competing talker) [13] and on the discrimination of minimal phonetic pairs [9, 14] resulted in generalization to untrained speech conditions. In contrast, multiday training on various acoustic [3] and visual [15, 16] discriminations appears quite specific to the trained features. For example, training on the discrimination of the duration of auditory intervals results in learning that is highly specific to the trained interval [5, 17, 18]. Even when two intervals are successfully learned, there is no transfer to an untrained temporal-interval that is adjacent (in length) to the two learned ones [18, 19]. A salient difference between the two types of studies is that training regimens for speech stimuli tend to incorporate a larger degree of stimulus variability across the training materials (e.g., multiple speakers and tokens) than non-speech training regimens which are characterized by massive repetition of acoustically identical stimuli throughout training, leading to the notion that stimulus variability during training contributes to the transfer of learning to untrained materials.

Although there is solid evidence that variability does facilitate speech learning [9–12], this is not always the case [20]. Rather, it seems that across-trial variability facilitates learning (facilitation is assessed by the extent of generalization to untrained tokens) when variability is introduced along a training-irrelevant dimension (e.g., speaker), but not when it occurs on the trained one (e.g., different phonetic contrasts). If this is the case, learning may not be dependent on the type of stimulus (speech, non-speech) or the training regimen (variable, non-variable), but rather on the relevance (or irrelevance) of the variable dimension. Indeed, several studies found that learning of phonetic discriminations improved with variable training, but in these studies variability was not introduced on the trained dimension (the specific minimal pair to be learned), but rather, along an irrelevant one (talker or word position of the practiced pair) [9–12]. On the other hand, the introduction of variability along a training relevant dimension seems to limit learning. For example, Nahum and colleagues [20] trained listeners on the discrimination of minimal word pairs in noise in one of two conditions. In one condition, the binaural cues that help the separation of speech and noise were delivered consistently across trials such that listeners received either diotic or dichotic stimulation for entire blocks of trials. In the other condition, diotic and dichotic stimuli were randomly mixed in each block. Whereas listeners in the first group improved with training, no learning was observed in the other group. Importantly, no transfer was observed in either group, demonstrating that inter-trial variability during practice does not necessarily widen the scope of generalization. This could also account for findings in the non-speech domain. For example, in a study on auditory duration discrimination [18] listeners practiced the discrimination of two auditory temporal intervals. Different base durations were presented in separate blocks of trials (no trial-by-trial variability), or randomly mixed within a block. Although both conditions were learned similarly, no generalization to untrained durations occurred with either. In the current study listeners were trained on the discrimination of minimal phonetic pairs (speech) and auditory durations (non-speech). Variability was introduced by randomly mixing different pairs or different base durations within a block of trials. Since these are training-relevant dimensions we predict similar patterns of learning following training with or without trial-by-trial stimulus variability (H1).

Even when successful, learning under variable conditions might be slower than learning under fixed stimulation conditions. Thus in the speech-in-noise discrimination study mentioned above [20], learning did occur when binaural cues were consistently interleaved across trials (diotic, dichotic, diotic, dichotic, etc.). Nevertheless learning was slower than in the group of listeners who received the two types of stimuli in different blocks. Similarly, trial-by-trial variability seems to slow the auditory learning of non-speech stimuli [7, 8]. For example, when trained on the detection of specific components of auditory patterns, listeners learned to detect the target stimulus faster when its location within the pattern remained constant across trials than when its location was variable [8]. The disruption of learning caused by stimulus variability during training is not confined to audition—studies in vision consistently show that stimulus variability slows or prevents learning altogether unless the stimuli are sufficiently different [21, 22]. Based on these, we now hypothesized that in this study trial-by-trial stimulus variability should slow learning (H2).

Despite the putative differences between the perceptual learning of speech and non-speech stimuli, the hypotheses presented so far are based on the tacit assumption that learning mechanisms are similar in the two domains. Given the traditional divide between ‘speech perception’ and ‘auditory perception’ [23] this assumption merits at-least some consideration/explanation. First, perceptual learning studies suggest that learning of speech and non-speech elements are governed by similar principles and constraints [20, 24, 25]. For instance, training listeners to categorize complex non-speech auditory patterns resulted in a pattern of neural changes highly similar to that induced by learning to categorize speech sounds [25]. Likewise, the interfering effects of stimulus variability on learning to discriminate speech-in-noise were similar to those induced by stimulus variability in visual perceptual learning [20]. Second, recent conceptualizations of phenomena that were considered unique properties of speech processing (e.g., talker normalization, categorical perception) emphasize the roles of general cognitive and perceptual mechanisms that support categorization and perceptual grouping [26, 27]. Within this framework we hypothesize that the effects of stimulus variability are similar in the learning of speech and non-speech elements once training and testing conditions are equated across the two types of stimuli (H3).

The current study was designed to test the three aforementioned hypotheses, that stimulus variability: (H1) does not facilitate learning if it occurs on the dimension on which training is administered; (H2) slows the learning process; and (H3) has similar influences on the perceptual learning of speech and non-speech sounds. To test these hypotheses, two *training modes* differing in the amount of trial-by-trial stimulus variability were created and administered to different groups of listeners using one of two *stimulus classes*—the discrimination of minimal phonetic pairs (‘speech discrimination’) and the discrimination of pure tones of different durations (‘duration discrimination’). Before and after training, half of the listeners who practiced each *stimulus class* and *training mode* were tested on both stimulus classes with stimulus variability, while the other listeners were tested on the same stimulus classes without variability. Pre- and post-test performance was also compared to the performance of untrained listeners. This design thus allows us to test the differential effect of both *training mode* (variable, non-variable) and *stimulus class* (speech, non-speech) on learning and generalization to the other stimulus class and other ‘variability condition’ (H1), and on learning rate (H2). A comparison of outcomes across stimulus classes and presentation modes allowed us to compare learning between the speech and non-speech domains (H3).

## Materials and Methods

### Ethics statement

All aspects of the study were approved by the ethics committee of the Faculty of Social Welfare and Health Sciences at the University of Haifa. Written informed consent was obtained from all participants prior to the first testing session.

### Participants

A total of 112 University of Haifa students (aged 20–35) participated in this study. Participants were native speakers of Hebrew and naïve to psychophysical testing. By self-report all had normal hearing and no history of language, learning or attention problems. Participants were compensated for the time devoted to the study.

The participants were divided into ten groups (see Table 1). Eight groups participated in the training program described below (initial n = 12 in each group). The trained groups differed on the trained stimulus class (speech stimuli, non-speech stimuli), the training mode (whether they trained with random or blocked stimuli), and the mode of pre- and post-testing they received (random, blocked). Two additional groups served as no-training control groups (initial n = 8 each). Listeners in these two groups participated in the pre- and post-test sessions, but differed in the mode with which they were tested (random or blocked).

**Table 1:** Training regimen (mode and stimulus class), testing conditions and final number of participants in each of the 10 study groups.

Group	Training regimen		Testing mode (pre- and post-test)
(final number of participants)	Training mode	Stimulus class	
1 (n = 9)	random	speech	random (speech and duration)
2 (n = 9)	random	speech	blocked (speech and duration)
3 (n = 6)	random	duration	random (speech and duration)
4 (n = 11)	random	duration	blocked (speech and duration)
5 (n = 8)	blocked	speech	random (speech and duration)
6 (n = 10)	blocked	speech	blocked (speech and duration)
7 (n = 8)	blocked	duration	random (speech and duration)
8 (n = 8)	blocked	duration	blocked (speech and duration)
9 (n = 6)	-----------	-----------	random (speech and duration)
10 (n = 6)	-----------	-----------	blocked (speech and duration)

Twenty-eight participants did not complete training and were excluded from data analysis. Data from three additional participants was lost due to technical errors. The data of 2 participants were removed during data analysis (see below), and thus data from a total of 81 listeners are reported (see Table 1 for the distribution of listeners across training and testing conditions).

### Experimental design

The experiment had three phases, a pre-test taken by all participants, a 9-sessions training phase completed by the participants of the 8 training groups and a post-test session completed by all participants. During the pre- and post-test sessions, conducted 4–8 weeks apart, listeners were tested on two stimulus classes, speech discrimination and duration discrimination (described below). The discrimination of stimuli within each class was tested in one of two modes (also described below), random or blocked, such that listeners in five groups (four training groups and one of the no-training groups) were tested with the random mode while listeners in the remaining five groups completed the blocked testing.

In an attempt to the mimic ‘real-life’ scenarios under which multisession training is likely to occur, a flexible training schedule was chosen for the current study. Trained listeners were asked to complete the study in 4–8 weeks, and undertake at-least one training session a week, with no further constraints. Prior to data analysis, training schedules were compared across the trained groups. Training schedules were similar across the eight trained groups in the current study (F(7,62) = 0.95, p = 0.48). On average listeners trained once every 2.4 days with a range of 2.1 (in duration trained listeners who practiced on a roving training mode but tested with blocked pre and post tests) to 2.7 days (in speech trained listeners who trained on the fixed mode but were tested with the roving mode). This suggests that any difference in training outcome between stimulus classes (speech vs. non-speech) or training modes (blocked vs. random) are not attributable to different training schedules between the trained groups.

All ten groups participated in pre- and post-test sessions in which they were tested on both speech discrimination and duration discrimination in one of two testing modes—blocked or random (never both). Two of these were untrained groups who participated in the pre- and post-test only. Four of the remaining groups were trained on the speech discrimination task (two with random training and two with blocked training). The final four groups were trained on the duration discrimination task (two with random training and two with blocked training). Therefore, as described in Table 1, during the pre- and post-tests, all trained groups were tested with the stimulus they trained with (speech/duration) as well as with the untrained stimulus (duration/speech). However, for half of the trained groups the training and test modes were identical (random or blocked) whereas the other half were trained with one mode but tested on the other. There were no conditions that were administered to all groups.

### Task and stimuli

Our goal was to determine whether variability differentially affects learning on two different classes of stimuli (speech and non-speech) when the two classes of stimuli are practiced with identical training regimens and tasks. Stimuli within each class were selected based on previous learning studies in each of the speech and non-speech domains. Therefore no attempt was made to match the stimuli across classes or to make the non-speech stimuli more speech-like in their acoustic properties.

Speech discrimination of four minimal phoneme pairs and auditory duration discrimination with four standard intervals were used in the current study, delivered in a 3-alternative forced-choice oddball task. On each trial, three stimuli were presented, two standard stimuli and one target stimulus. Listeners were instructed to determine which of the three presented stimuli was different from the others. Target stimuli varied adaptively in a 3-down/1-up staircase procedure in 40-trial runs. A multiplicative staircase was used. Step size was two until the fourth reversal and 1.41 thereafter. Adaptive runs were randomly interleaved and administered in blocks of 160 trials. Visual feedback was provided throughout the entire experiment for both correct and incorrect responses.

**Speech discrimination.** Four pairs of synthetic speech tokens (described by Moore et al. [14]) were presented either randomly or blocked (see below): [bee]-[dee], [da]-[ga], [ma]-[na], and [sa]-[sha]. Each pair formed the endpoints of a 96 item continuum. On each trial, two identical standard stimuli and one target stimulus were presented. Standard stimuli were defined as one endpoint of each continuum ([bee], [da], [ma], [sa]). Target stimuli were adapted from the other endpoint and towards the standard.

**Duration discrimination.** Four standard stimuli were used in this task, presented either randomly or blocked (see below)– 50 ms, 100 ms, 200 ms and 350 ms. All stimuli were 1-kHz pure tones. On each trial two identical standard tones and one target tone were presented with an 800-ms inter-stimulus-interval. Target stimuli were adapted from a starting point of 1.5 times the duration of the standard, towards the duration of the standard stimulus.

### Pre- and post-testing sessions

**Testing modes—random and blocked.** Each task had two testing *modes*—random and blocked. In random testing the four different stimuli used for each task were randomly mixed (roved) within each block of 160 trials. In blocked testing only one type of stimulus was presented within a block. Each group of listeners was tested on one of the modes and trained on either one or the other of the modes such that the effects of training on performance could be tested as a function of both training and testing modes (see Table 1), without confounding the two.

**Stimulus classes—speech and duration.** During the pre- and post-test sessions listeners were tested on both the duration discrimination and the speech discrimination described above in separate blocks (160 trials each). Each stimulus was presented for a single run of 40 trials. Participants tested on random mode completed a single block of 160 trials per task, comprised of randomly mixed runs of the 4 stimuli (of the same class). Participants tested on the blocked mode completed a 4 single blocks of 40 trials per task (8 in total).

### Training regimens

Each group of trained listeners participated in nine training sessions of 640 trials (lasting approximately 45 minutes) each, divided into four 160-trial blocks. Each group practiced either speech or duration discrimination. Likewise, each group practiced only a single mode, either random or blocked. Each block consisted of 4 independent, adaptive runs of 40 trials mixed within the block on a trial-by-trial basis. In random training sessions, each run consisted of one of the four stimuli, randomly mixed with runs of the other three stimuli, such that within a block of 160 trials listeners encountered 40 presentations of each target stimulus. In blocked training sessions, all 4 runs in each block consisted of only one of the four stimuli, so that listeners practiced all four stimuli in consecutive blocks. The order of the different stimulus-blocks was randomized across participants but kept constant across the training sessions of each participant.

### Data analysis

Many listeners were very poor at the start of training, especially under the random conditions. Since both stimulus classes were upper-bound (not allowing stimuli to become too long in the duration discrimination task, and bound by the end-points of the syllable continua in the speech task), there were no viable reversals to use in blocks where performance failed to reach 79% correct at the greatest difference. Discrimination thresholds were therefore calculated as the geometric average of the last 20 trials per adaptive run, which did not differ greatly from the 79% correct thresholds obtained on those blocks where there were a sufficient number of viable reversals. For each participant, a single threshold was obtained for each stimulus for the pre- and post-tests, and four thresholds per stimulus for each of the training sessions. Log-transformed thresholds were used for statistical analysis because the adaptive step was multiplicative.

Prior to statistical analysis all individual data were visually inspected, and the data of two listeners who performed at floor level consistently throughout training and testing were excluded. The performance of those two listeners was consistently at the poorest possible level of performance afforded by the staircase procedure used. In addition, the duration discrimination data from the post-test of an additional participant were excluded because this listener performed all 4 post-test conditions at a floor level, despite a reasonable performance at pre-test and during training.

### Pre- to post-test learning

**Fig 1:**
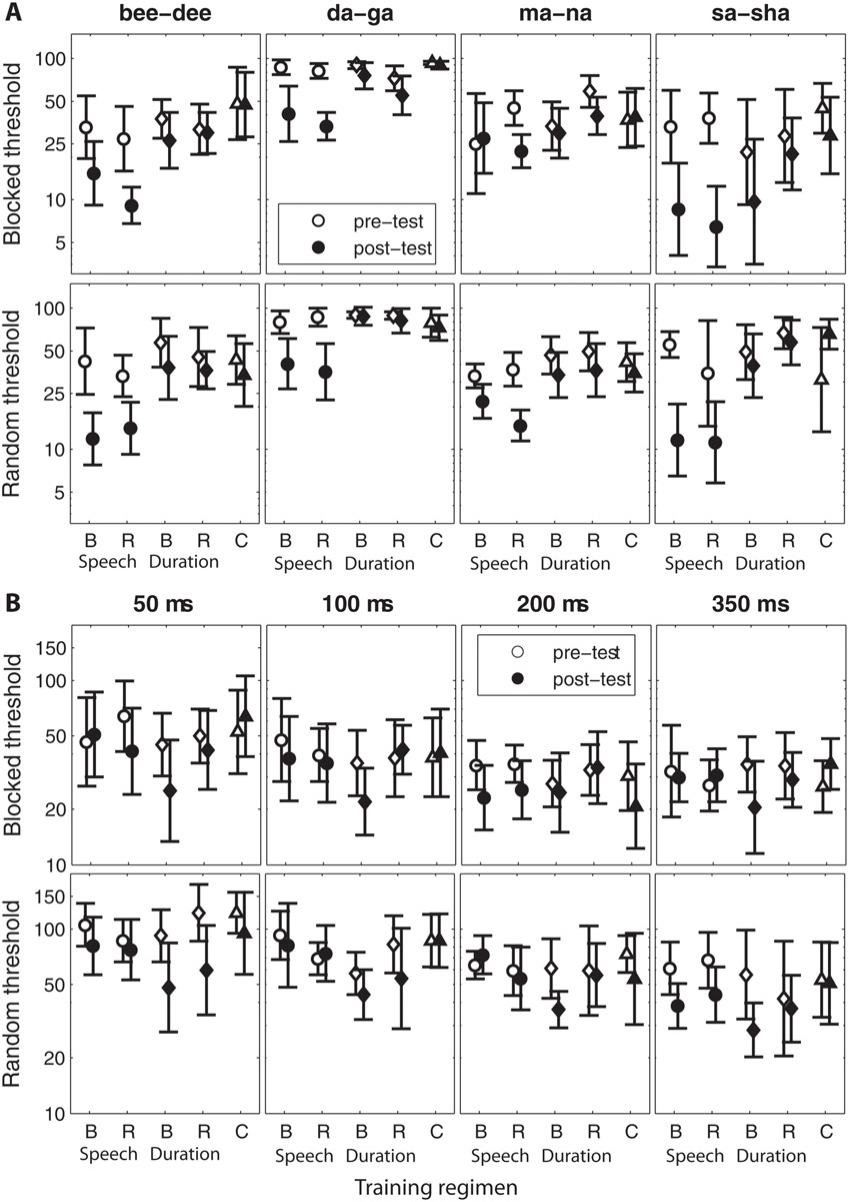
Pre- and Post-test thresholds for each training stimulus, stimulus class and testing mode. **(A)** Pre-test (empty symbols) and post-test (full symbols) thresholds on the speech discrimination task for each phoneme minimal-pair stimulus. Thresholds are plotted as the phoneme number (on the continuum between 1 and 96), tested on the blocked condition (top panels) and random mode (bottom panels). Five training groups (shown from left to right on the x-axis of each panel) were tested on each mode: Blocked (B) and Random (R) Speech, Blocked (B) and Random (R) Duration, and untrained Controls (C). Note that the top and bottom panels denote different training groups (10 groups in total; see Methods for details). All error bars denote 95% confidence intervals. **(B)** Pre- and post-test thresholds on the duration discrimination task for each reference stimulus duration, plotted as per-cent of the reference duration. Top panels show performance on the blocked tests, while the bottom panels show performance on the random test for the same groups as (A).

As shown in Fig. 1 (and confirmed with a one-way ANOVA), mean performance was similar between the different groups across individual tasks and stimuli. Therefore, speech- and duration-discrimination thresholds were submitted to two omnibus ANOVAs with time and stimulus as within-listener factors and testing mode (blocked, random), training mode (blocked, random) and stimulus class (speech, duration) as between-listener factors. This analysis revealed no significant interactions between testing mode (random, blocked) and any of the other factors (or interactions between factors), suggesting that the way listeners were tested prior to training had no influence on any of the other effects of interest (time, training mode and stimulus class). We therefore analyzed the effects of training with a series of ANOVAs on the pre- and post-test thresholds in the two different modes (random, blocked), with time (pre-test, post-test) and individual stimulus ([bee]-[dee], [da]-[ga], [ma]-[na] and [sa]-[sha] or 50 ms, 100 ms, 200 ms and 350 ms) as within listener factors and training mode (random, blocked) and stimulus class (speech, duration) as between-listener factors. Main effects of time were taken as evidence for training induced learning, while effects of training mode and stimulus class were used to determine whether these modified learning. Main effects of stimulus are reported, but not further considered or analyzed since the differences between the stimuli making each of the tasks are beyond the scope of the current study. Greenhouse-Geisser corrections were used in all cases where data were not spherical.

To determine whether pre- to post-test changes in the trained groups are attributable to learning, the amount of pre- to post-test improvements on the different testing modes were compared between trained and untrained listeners using a series of contrasts. Amount of improvement was quantified for each listener and stimulus class by converting the four individual thresholds (speech: [bee]-[dee], [da]-[ga], [ma]-[na] and [sa]-[sha]; duration: 50 ms, 100 ms, 200 ms and 350 ms) to Z-scores (relative to the relevant pre-test means). Individual Z-scores were then averaged across the four pre- and post-tests for each stimulus class, yielding a total of four Z-scores per participant (pre speech, pre duration, post speech, post duration). Post-test Z-scores were then subtracted from pre-test scores and the differences were compared across groups.

#### Training-phase learning

A mean learning curve was calculated for each participant by averaging over all of the daily thresholds. Individual regression lines were then fitted to the session average and the slopes of these lines were submitted to statistical analysis to determine whether training mode and stimulus class had differential effects on learning.

## Results

### Learning and transfer between the pre- and post-tests

Mean group discrimination thresholds are shown in Fig. 1, across all stimuli, as a function of stimulus class, training mode and test mode. A visual inspection of the data presented in Fig. 1A suggests that with the exception of the [ma]-[na] blocked condition, speech discrimination in both test modes (blocked, and random, shown on the top and bottom rows respectively) improved substantially following training on either of the two modes of the speech task (blocked and random). As for duration discrimination, Fig. 1B suggests pre- to post-test changes were small and inconsistent across stimuli, but these were not specific to either class or training mode. As explained in the data analysis section of the Methods, these observations were confirmed statistically by submitting the pre- and post-test speech and duration thresholds to a series of ANOVAs with time (pre, post) and stimulus ([bee]-[dee], [da]-[ga], [ma]-[na] and [sa]-[sha] or 50-ms, 100-ms, 200-ms and 350-ms) as within-listener factors and training mode (blocked, random) and stimulus class (speech, duration) as between-listener factors. The outcomes of these analyses are reported in the following sections. Note that because we were not specifically interested in the differences between the trained stimuli within each class of stimuli, interactions involving stimuli were not explored further and are not reported.

### Learning and transfer on the speech task

**Fig 2:**
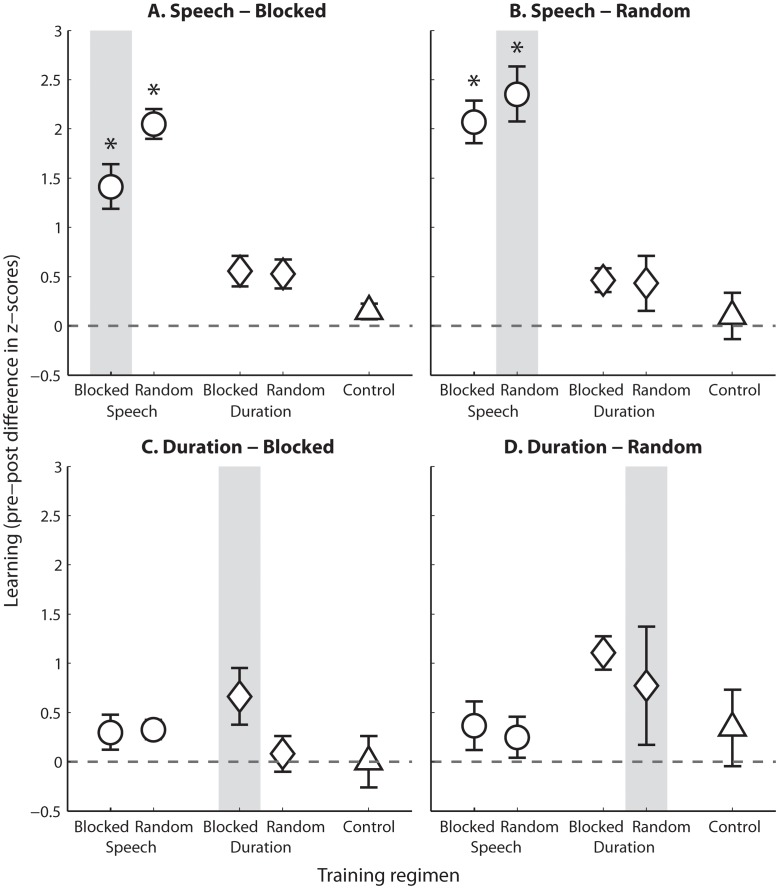
Learning on each stimulus class and testing mode. Learning is shown as the difference between pre- and post-test thresholds. Individual z-scores were calculated with respect to the aggregate pre-test data across all participants tested on each stimulus class and mode (see Methods), and are thus plotted in units of standard deviation from pre-test scores (dashed line at 0) for each group. Data are shown for Blocked **(A)** and Random **(B)** Speech, and Blocked **(C)** and Random **(D)** Duration, in the groups trained on Blocked and Random Speech, Blocked and Random Duration, and untrained Controls (denoted on the x-axis of each panel). The grey box in each panel indicates the group for which training and testing was on the same regimen. Asterisks demarcate significant learning compared to the Control group (after correction for multiple comparisons, see Table 2). Error bars denote 95% confidence intervals.

Performance on the blocked speech tests (Fig. 2A) was significantly influenced by time (F(1,34) = 60.6, p < 0.001, η^2^_p_ = 0.64) and individual stimulus (F(2.2, 74.1) = 42.0, p < 0.001, η^2^_p_ = 0.55), with a marginal effect of stimulus class (F(1,34) = 3.68, p = 0.063, η^2^_p_ = 0.10). Suggesting that learning did not transfer across stimulus classes, a strong time × class effect was also observed (F(1,34) = 14.5, p < 0.001, η^2^_p_ = 0.30). Indeed, listeners who trained on either mode of the speech task improved their speech discrimination thresholds between the pre- and post-tests significantly more than controls, but listeners who practiced the duration task did not (see Table 2 and Fig. 2A). No other main effects or interaction terms were significant.

**Table 2:** Pre- to post-test learning vs. untrained controls in Z-score units.

		Speech		Duration	
		Random	Blocked	Random	Blocked
Training regimen	Random speech	2.25***	-1.90***	-0.09	0.33
	Random duration	0.33	0.38	0.43	0.08
	Blocked speech	1.97***	1.27**	0.02	0.30
	Blocked duration	0.36	0.41	0.76	0.66

Statistical significance was determined using a contrast between untrained listeners and each of the trained groups. Contrast values are significant at

***p < 0.001,

**p < 0.01.

All significant contrasts remain significant with Bonferroni corrections.

Three factors significantly influenced speech discrimination in the random testing mode (Fig. 2B)—time (F(1,27) = 56.8, p < 0.001, η^2^_p_ = 0.68), individual stimulus (F(2.3, 58.8) = 30.6, p < 0.001, η^2^_p_ = 0.53) and training stimulus class (F(1,27) = 22.7, p < 0.001, η^2^_p_ = 0.46). The significant effect of time suggests that pre- to post-test learning occurred in this condition. The amount of learning was however modified by the training stimulus class (time × class interaction, F(1,27) = 23.4, p < 0.001, η^2^_p_ = 0.46) confirming that listeners that trained on the speech task improved more than listeners who trained on the duration task between the pre- and post-test (see Fig. 1A, bottom panels and Fig. 2B). No other main effects or interaction terms were significant, suggesting that random training was not more likely to improve speech discrimination than blocked training. Consistent with the finding that learning was specific to the trained stimulus class, but not to the training mode (random or blocked), a contrast analysis suggests that only speech trained listeners improved significantly more than controls between the pre- and post-test on the random speech task (Fig. 2B, and see Table 2 for contrast values and significance).

Together, these data suggest that in general, improvements in speech discrimination were specific to speech training, regardless of whether the training was delivered in a random or blocked fashion. That no influence was found for the random training regimen suggests that, consistent with our initial hypothesis (H1), in the current study there was no advantage for random over blocked training for learning speech discrimination. We also found no evidence of significant transfer from duration training to speech discrimination regardless of training or testing modes.

### Learning and transfer on duration discrimination

As shown in Fig. 1B (top panels) and in Fig. 2C, learning on the blocked duration tests was weakest compared to the other learning effects (main effect of time: F(1,34) = 5.37, p = 0.027, η^2^_p_ = 0.14). Although stimulus also affected performance in this task (F(2.4, 80.4) = 16.3, p < 0.001, η^2^_p_ = 0.32) no other main effects or interaction terms were significant suggesting that neither the trained stimulus class (speech, duration) nor the training mode (random, blocked) had any discernible effects on learning in this condition. Likewise, duration trained listeners did not improve between the pre- and post-tests more than untrained controls (Table 2 and Fig. 2C).

Duration discrimination in the random test mode (Fig. 1B, bottom panels and Fig. 2D) changed significantly as a function of both time (F(1,26) = 9.50, p = 0.005, η^2^_p_ = 0.27) and stimulus (F(3,78) = 21.5, p < 0.001, η^2^_p_ = 0.45), and marginally so as a function of the trained stimulus class (F(1,26) = 3.25, p = 0.083, η^2^_p_ = 0.11). No other main effects or interaction terms were significant. Consistent with the relatively small effect size of the pre- to post-test change on the random duration discrimination test, neither of the duration trained groups learned significantly more than controls (Fig. 2D and Table 2).

Together these data suggest that for the blocked as for the random test modes, learning was sensitive and specific to the trained stimulus class (duration vs. speech), but not to the training mode (random vs. blocked), again, consistent with our initial hypothesis (H1) concerning the effect of the training regimen. These data also suggest that in this study speech discrimination was learned more readily than duration discrimination.

### Training-phase learning

**Fig 3:**
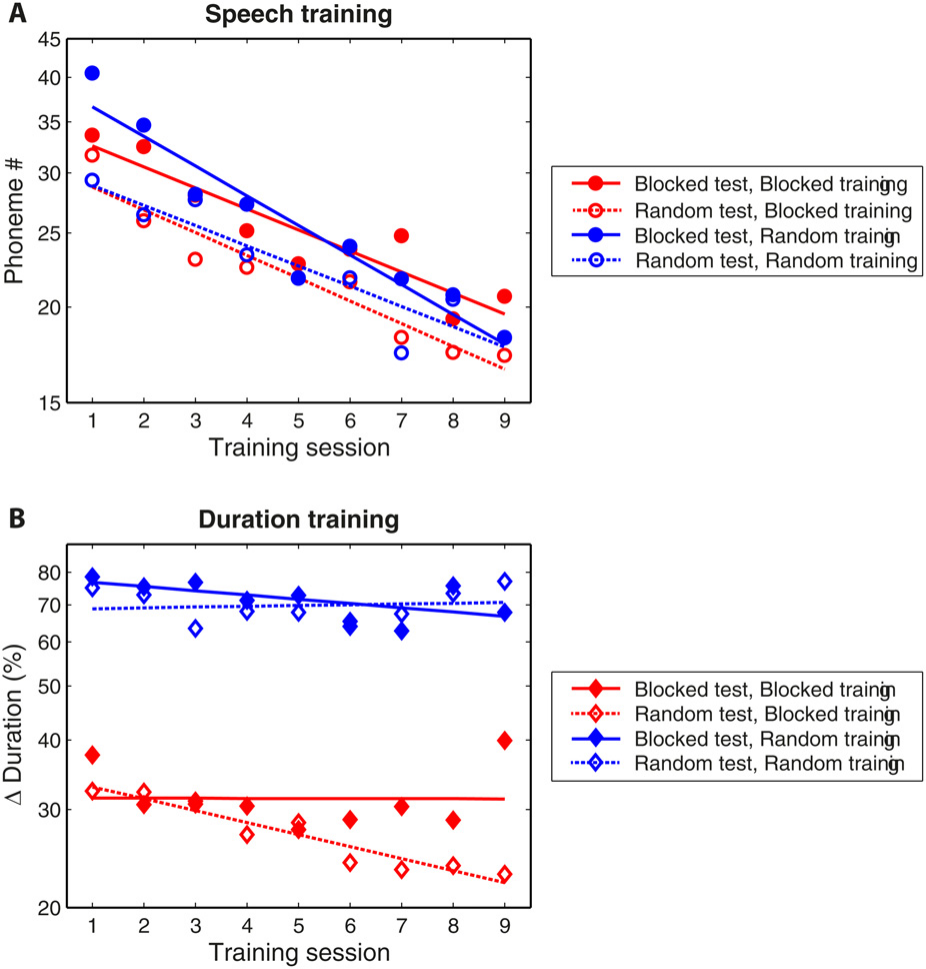
Group mean learning curves for all training regimens. Group mean learning curves for the groups trained on Blocked (red) and Random (blue) Speech **(A)** and Duration **(B)**. Dotted and full lines are the regression lines fitted to the mean thresholds averaged across stimuli and participants for each training session, in the groups tested on the random and blocked mode, respectively. Error bars are not shown because they overlap substantially.

Mean daily discrimination thresholds (pooled across the trained stimuli) are shown in Fig. 3. The fitted regression lines suggest that robust learning (negative slopes) occurred during speech discrimination training across the different groups that trained on speech discrimination, irrespective of training or testing mode (Fig. 3A). This was clearly not the case for listeners who practiced duration discrimination in which learning, if any, appeared to occur with blocked training only (Fig. 3B). To determine whether the training mode (random, blocked), testing mode (random, blocked) and trained stimulus class (speech, duration) influenced the rate of learning during the training phase, individual regression slopes were submitted to a 2 (testing modes) × 2 (training modes) × 2 (stimulus class) ANOVA. Only the stimulus class significantly impacted the slopes (F(1, 68) = 14.5, p < 0.001, η^2^_p_ = 0.19), suggesting that training on speech discrimination yielded greater learning than training on duration discrimination. Indeed, the mean slopes and their confidence intervals, shown in Table 3, were significantly negative in all the groups that practiced speech discrimination but only in one of the four groups of listeners who practiced duration discrimination (those who practiced on the blocked mode but tested on the random mode). Thus in contrast to our initial hypothesis (H2), when learning occurred during the training phase, it was not slower with random than with blocked training.

**Table 3:** Mean group slopes of the regression lines fitted to individual learning curves [with 95% confidence intervals].

		Pre- and post-test	
		Random	Blocked
Training regimen	Random speech	-0.027 [-.046, -.007]	-0.039 [-.062, -.016]
	Random duration	0.001 [-.032, .035]	-0.008 [-.026 0.011]
	Blocked speech	-0.029 [-.049, -.016]	-0.028 [-.048, -.008]
	Blocked duration	-0.021 [-.035, -.008]	-.000 [-.018, 0.18]

## Discussion

The major outcomes of the current study were that (1) the discrimination of minimal phonetic contrasts was learned equally with random and blocked training regimens; (2) speech learning with either one of the regimens fully generalized to the other; (3) no learning was observed on the duration discrimination task with either a random or a blocked training regimen (see Fig. 2 for a visual summary). These findings are consistent with the idea that variability along a training-relevant dimension is not conducive to learning (initial hypothesis H1), but not with the hypothesis (H2) that trial-by-trial variability slows learning. In contrast to what we expected based on previous studies [18, 19], no significant learning was observed on duration discrimination with either roving or blocked training. Therefore, the data neither confirmed nor disconfirmed our hypothesis (H3) that the effects of variability are similar in the learning and generalization of speech and non-speech elements. In the following sections we discuss the potential causes for the lack of learning (and generalization) on duration discrimination, as well as the different natures of speech and non-speech stimuli which could have made speech stimuli more learnable and more robust to stimulus variability.

### Why did listeners learn speech, but not duration discrimination? Methodological Considerations

The current finding that training on duration discrimination (either blocked or roving) yielded no learning, was surprising because previous reports led us to expect learning on the blocked regimen (e.g., [5, 19]), if not on both regimens [18]. Nevertheless, the current study was not an exact replication of any previous study, and several methodological factors could account for this failure and are discussed below—the random-like nature of our blocked regimen, the number of training trials per condition, the number of different stimuli trained or the spreading of training over a longer period than in previous studies.

First, although blocked trained listeners practiced each of the four stimuli on separate blocks of trials, each block was comprised of four randomly interleaved runs. Therefore, although the same reference stimulus appeared on each and every trial, adaptation of the target stimuli was conducted separately for each run. This could have resulted in uneven changes in the values of the target stimuli across runs, leading to the presentation of very different values of the target stimulus across trials. Therefore, although listeners heard only one stimulus per block, they may have actually faced a more variable situation than listeners in previous studies in which duration discrimination learning was observed [5, 18, 19], leading to a disruption of the learning process. Random presentation should not have precluded learning entirely [7, 18]. However, the mixed stimuli in our blocked conditions differed from both these studies in that they were more similar to each other (e.g., 100–200 ms in the 100 ms condition) than the mixed stimuli in the Karmarkar and Buonomano [18] study that differed in both frequency and base duration (e.g., around 100 ms and around 350 ms). Likewise, large frequency differences between base frequencies did not disrupt frequency discrimination learning (for most listeners), while small differences did [7]. It is also consistent with the notion arising from studies in visual perceptual learning that have repeatedly shown that stimulus variability disrupts learning when the varied stimuli are similar, but not when they are highly distinctive [21, 22, 28].

Second, the amount of training provided on each of the stimuli (160 trials of each stimulus on each training session) might have been sufficient for speech discrimination learning, but too small to initiate learning on duration discrimination. Indeed, previous studies in auditory perceptual learning suggest that the minimal number of practice trials required to initiate learning varies greatly across different tasks [29]. For example, it has been previously shown that although 360 training trials/session were sufficient to yield learning on temporal-interval duration discrimination, this number was not sufficient to yield learning on a frequency discrimination task with the same stimuli [29]. Nevertheless, other studies on frequency discrimination showed that the number of training trials also changes as a function of the particular stimulus used. Whereas 360 trials/session were not sufficient to initiate frequency discrimination learning when the stimuli were 15-ms tone pips separated by silent intervals [29], 100 trials were adequate to initiate learning with longer (100-ms) stimuli [30]. It is quite possible that task difficulty is the critical factor in determining the amount of training required to initiate learning, and that duration discrimination presents a greater challenge perceptually than the speech continua used here.

Third, whereas previous studies of duration discrimination used training regimens that mixed two different stimuli [18, 19], ours employed four different base durations. However, we think it unlikely this is responsible for eliminating the learning, as frequency discrimination learning was shown to slow rather than disappear entirely when five base frequencies were used [7].

Finally, although the total amount of training provided to listeners in the current study was similar to the amount that was found previously to yield learning [19,29], the training schedule in the current study may have been different than that of previous studies [5, 18, 19, 29]. Whereas in previous studies (when reported) training typically occurred on consecutive days with larger gaps only during weekends [19, 29], here greater spacing of the training sessions was possible. Nevertheless, listeners typically trained every two to three days (see Methods). Therefore, although we cannot rule out session spacing, we think it unlikely that that lack of learning here was an outcome of the more flexible training schedule we allowed.

Other factors that could have contributed to the lack of learning—the final number of trained listeners, between-listener variability and number of excluded data points—were similar to those reported in previous studies [19, 29]. This suggests that the lack of learning did not result from the current sample being unusual or too variable.

Whatever caused the lack of learning on duration discrimination makes it impossible for us to draw any conclusions regarding the effects of variability on this type of learning. Although we cannot rule out that the methodological factors considered above were responsible for the lack of learning on duration discrimination in the current study, it is nevertheless revealing that non-speech learning may be more sensitive to those variables than speech learning. In the following section we discuss why this might be the case, although determining whether our proposal is indeed correct requires further studies.

### Variance, invariance and the (different) nature of speech and non-speech stimuli

The current data strongly support the notion that, at least as tested here, speech discrimination learning is more robust to interference than non-speech auditory learning. As discussed below, we tentatively propose that despite the methodological considerations noted above, the different nature of the syllables and the non-verbal stimuli led both to the divergent learning outcomes and to the similarity of the outcomes of random and blocked speech discrimination training. Specifically, we propose that the categorical nature of the speech stimuli may have made them more distinctive and thus more easily learned and more immune to the interference induced by stimulus variability than the non-speech stimuli which appear to form a continuous dimension.

### The ability to categorize/label speech makes it easier to learn

Random or blocked, four different speech stimuli and four different non-speech stimuli were used as reference stimuli in this study. Whereas the (minimal) syllable pairs differed on different acoustic phonetic features (e.g., place and manner of articulation) which would have made the category endpoints easily discriminable for naïve listeners, this may not have been the case for duration discrimination. Naïve performance on the duration discrimination task (Fig. 1B, empty symbols) supports this idea. For example, mean naïve thresholds on the roving condition were in the order of 100% of the reference duration. This implies that the target tones representing each of the different stimuli initially overlapped adjacent base durations, making the four different stimuli less discernible than the speech stimuli, thereby disrupting both discrimination and learning. On the other hand, the four reference speech stimuli ([da], [bee], [ma], [sa]) were taken from different phonetic categories, which should have made them easily discernible (e.g., /b/ and /m/ are both bilabial but one is plosive and the other is nasal, while /s/ is a fricative and [ee] is a different vowel category than [a]). Indeed, when only two, more distinct, base durations were roved [31], naïve thresholds were much lower than in the current study.

That duration learning was disrupted by the similarity between the stimuli even in the blocked conditions is consistent with our previous proposal that the ability to categorize stimuli to distinct perceptual categories is conducive to learning, especially under variable conditions [4, 32]. By this account the difference between learning speech and non-speech discrimination in this study was driven by the greater role of categorization in the auditory perception of speech than non-speech (for review, see [26]). Indeed, whereas endpoint stimuli in our speech tasks could have been easily assigned to four distinct categories ([bee], [sa] etc.), this was not the case for the stimuli in the duration task which appear to form a continuous, rather than a categorical dimension [33]. Therefore, on either random or blocked conditions, it would have been quite easy for listeners to (implicitly) identify the reference stimulus in the case of speech, but harder to do so in the case of the non-speech stimuli. Rather, in the case of duration they would have to resort to a true comparison across the lengths of the stimuli presented on each trial, which could have interfered with task performance, and subsequently with learning [34]. Similarly, it has been proposed that perceptual learning is strongly constrained by perceptual constancy [35]. By this account, the ability to classify variable sensory inputs to perceptually invariant units guides learning. Since this classification would have occurred more readily for the speech than for the non-speech stimuli, speech discrimination was learned, but duration discrimination was not. Adini et al. [12] made an even stronger claim—that variable training (roving) disrupts learning when discrimination necessitates exact knowledge of the stimulus. Thus, while in speech it is clear what the stimulus is (i.e., at least the endpoint reference stimulus can be clearly labelled), not so in duration, which is a relative quantity.

### The ability to categorize speech makes it less sensitive to variability

In contrast with previous speech learning studies [9–12], we observed no differences between the outcomes of random and blocked training when it comes to speech learning. Rather, the learning curves on the two conditions were nearly overlapping (Fig. 3A). Furthermore, training on either the random or the blocked mode similarly generalized to the other, untrained mode (Fig. 2A and B, circles). We argue that this was the case because the speech stimuli could be categorized. If this is indeed the case, the ability to categorize non-speech sounds should also allow learning when variability across training-irrelevant dimensions is present (as these do not involve a category change). Although here we saw no learning with the non-speech stimuli and did not test for generalization to untrained tokens, previous studies nevertheless demonstrated that auditory perceptual learning can transfer across training-irrelevant dimensions [7, 18, 19, 36]. For example, successful learning on auditory duration discrimination transferred across tonal frequencies (a training-irrelevant dimension), but not across durations (the trained dimension), although it is not clear how training-set variability affects this pattern [18, 19].

Finally, taken together our data suggest that concurrently training several stimuli is more efficacious when they can be categorized, or are sufficiently distinct from one another. This has implications for the design of perceptual training programmes, especially those using mixed training regimens.
